# Droplet Friction on Superhydrophobic Surfaces Scales With Liquid‐Solid Contact Fraction

**DOI:** 10.1002/smll.202405335

**Published:** 2024-09-17

**Authors:** Sakari Lepikko, Valtteri Turkki, Tomi Koskinen, Ramesh Raju, Ville Jokinen, Mariia S. Kiseleva, Samuel Rantataro, Jaakko V.I. Timonen, Matilda Backholm, Ilkka Tittonen, Robin H.A. Ras

**Affiliations:** ^1^ Department of Applied Physics Aalto University P.O. Box 15600 Espoo 02150 Finland; ^2^ Center of Excellence in Life‐Inspired Hybrid Materials (LIBER) Aalto University P.O. Box 15600 Espoo 02150 Finland; ^3^ Department of Electronics and Nanoengineering Aalto University P.O. Box 13500 Espoo 02150 Finland; ^4^ Department of Chemistry and Materials Science Aalto University P.O. Box 16100 Espoo 02150 Finland; ^5^ Department of Electrical Engineering and Automation Aalto University Maarintie 8 Espoo 02150 Finland

**Keywords:** contact angle hysteresis, droplet friction, liquid–solid contact fraction, superhydrophobicity

## Abstract

It is generally assumed that contact angle hysteresis of superhydrophobic surfaces scales with liquid–solid contact fraction, however, its experimental verification has been problematic due to the limited accuracy of contact angle and sliding angle goniometry. Advances in cantilever‐based friction probes enable accurate droplet friction measurements down to the nanonewton regime, thus suiting much better for characterizing the wetting of superhydrophobic surfaces than contact angle hysteresis measurements. This work quantifies the relationship between droplet friction and liquid–solid contact fraction, through theory and experimental validation. Well‐defined micropillar and microcone structures are used as model surfaces to provide a wide range of different liquid–solid contact fractions. Micropillars are known to be able to hold the water on top of them, and a theoretical analysis together with confocal laser scanning microscopy shows that despite the spiky nature of the microcones droplets do not sink into the conical structure either, rendering a diminishingly small liquid–solid contact fraction. Droplet friction characterization with a micropipette force sensor technique reveals a strong dependence of the droplet friction on the contact fraction, and the dependency is described with a simple physical equation, despite the nearly three‐orders‐of‐magnitude difference in liquid–solid contact fraction between the sparsest cone surface and the densest pillar surface.

## Graphical abstract

The general understanding is that droplet friction on superhydrophobic surfaces is affected by liquid–solid contact fraction. This work experimentally quantifies this relation for the first time. State‐of‐the‐art droplet friction measurements on micropillar and ‐cone surfaces with significantly different contact fractions show that droplet friction depends similarly on the contact fraction on both surface types despite the large difference in the measured droplet friction.

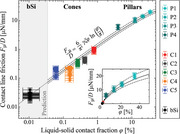

## 1 Introduction

The ability to control how water wets a solid surface enables applications like self‐cleaning,^[^ ^1^ ^]^ anti‐icing,^[^ ^2^, ^3^ ^]^ anti‐fogging,^[^ ^4^, ^5^ ^]^ and drag reduction.^[^ ^6^, ^7^, ^8^ ^]^ Common for these examples is that they all require minimal adhesion or friction between water and the surface, which usually is achieved by making the surface hydrophobic. Barthlott et al. found in the 1970′s that extreme hydrophobicity is achieved when the surface has microscopic roughness combined with hydrophobic surface chemistry, e.g., lotus leaves have ca. 10 µm sized papillae covered with hydrophobic wax.^[^ ^9^ ^]^ On such surfaces, water wets only the vertices of the microstructures (i.e., remains in Cassie wetting state), which leads to minimal contact and adhesion between water and the surface (i.e., minimal liquid–solid contact fraction, LS–CF), enabling water to roll off from the surface even at minor tilt angles.^[^ ^10^ ^]^ This phenomenon is termed superhydrophobicity and is usually defined as a water contact angle exceeding 150° and a droplet roll‐off angle less than 5° or 10°.

**Figure 1:**
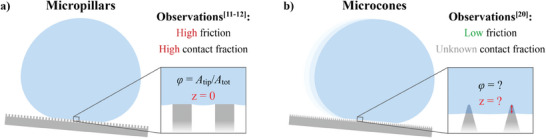
a) On micropillar surfaces, droplets can remain atop the pillars without observable sinking (*z*  =  0), and therefore the LS–CF (φ) can be calculated based on pillar width and spacing. The resulting friction is relatively high.^[^ ^11^, ^12^ ^]^ b) The liquid–solid contact fraction on microcone surfaces is more difficult to determine since droplets may sink below the cone tip level (*z* ≥ 0). Nevertheless, the resulting friction can be very low at least for certain surfaces with micrometer sized cones.^[^ ^20^ ^]^

Over the years, numerous artificial superhydrophobic surfaces have been developed based on various nano‐ and microstructure designs.^[^ ^11^, ^12^, ^13^, ^14^, ^15^, ^16^, ^17^, ^18^, ^19^ ^]^ One notable design type is a periodic array of micropillars (see **Figure** **1**a), as it has provided a simple model design to study how various geometrical parameters affect different wetting properties of droplets in the Cassie state. Earlier works for example by Reyssat & Quéré and Qiao et al. show that water droplet roll‐off angle and sliding friction become smaller when the LS–CF is reduced by tuning the micropillar size and spacing, yet the lowest LS–CF they considered was still fairly high (≈5%) having substantial contact angle hysteresis beyond 10°.^[^ ^11^, ^12^ ^]^ Another notable design type is nano‐ and micro‐sized cones, spikes, or needles (see Figure 1b), in particular, due to the extremely low droplet sliding friction shown for this surface design.^[^ ^20^ ^]^ These surfaces have much smaller tip vertices than micropillars, suggesting that the extremely small sliding friction of the droplet is due to a small LS–CF. However, an open question remains on how droplets actually “sit” on the sharp tips; whether they can remain on the tip vertices or whether they sink partly inside the structure. Thus, their LS–CF has either remained unknown or has been estimated via contact angles from the Cassie‐Baxter equation,^[^ ^21^, ^22^ ^]^ which may be inaccurate due to theoretical assumptions behind that equation^[^ ^23^ ^]^ and difficulties of accurate contact angle measurements of superhydrophobic surfaces,^[^ ^24^, ^25^ ^]^ and no work for determining the relationship between LS–CF and droplet sliding friction has been performed earlier on conical surface structures.

In this work, we quantify in detail the relationship of LS–CF and droplet sliding friction (specifically contact line friction, CLF) by investigating the friction on both regular micropillar and microcone surfaces that are superhydrophobic. We first theoretically investigate how water droplets sit on the microcone surfaces and note that droplets prefer to remain atop the cone tips as long as the cones are densely enough spaced. In addition, we use confocal laser scanning microscopy (CLSM) imaging to determine the height of the air layer (called plastron) trapped between the solid surface and the droplet and observe that plastron height approximately equals cone height determined with electron microscopy, which supports the theoretical prediction that droplets remain atop the cone tips. This allows calculation of the LS–CF of droplets on cone surfaces based on the regular surface geometry, revealing that LS–CF as low as 0.06% is enough to support droplets atop the cone tips. Water on such surfaces is extremely mobile and it is practically impossible to determine droplet sliding friction via sliding angle or contact angle hysteresis measurements due to their limited accuracies,^[^ ^24^, ^25^ ^]^ and more advanced cantilever‐based wetting characterization techniques are needed.^[^ ^20^, ^26^, ^27^, ^28^, ^29^, ^30^, ^31^, ^32^ ^]^ For that reason, we characterize the sliding friction with the micropipette force sensor (MFS) technique^[^ ^20^, ^28^, ^30^, ^33^ ^]^ and show that the droplet CLF on the microcone surfaces is one to two orders of magnitude lower than on micropillar surfaces. Yet, the droplet CLF on both surface textures scales similarly with the LS–CF regardless of the large, nearly three orders of magnitude difference in the LS–CF. This relation between droplet friction and liquid–solid contact fraction can be described via a simple physical equation. We find that our results help understanding droplet mobility on conical microstructures and thus how microcone surfaces would perform under various applications that benefit from minimal contact between water and the surface.

## 2 Results and Discussion

**Figure 2:**
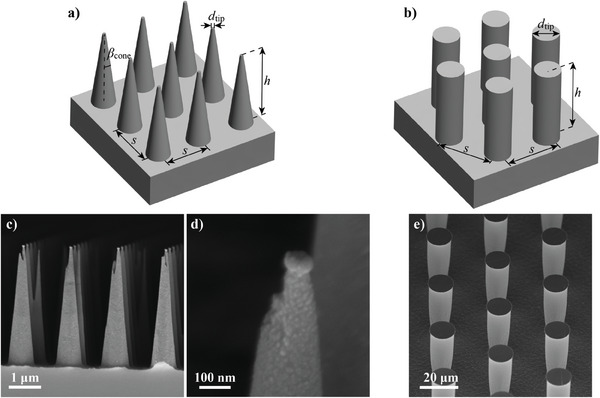
Schematics of a) microcone and b) micropillar array geometries. *d*_tip_ is tip diameter, *h* is height, and *s* is spacing between the pillars/cones, measured from cone center to center, and *β*_cone_ is the cone tip half angle. c) SEM image of the cone surface C2. d) High magnification of a typical cone tip, image from surface C2. e) SEM image of the P3 pillar surface. SEM images of the other cone and pillar surfaces are in Figure S1,S2 (Supporting Information).

We prepared five microcones (labeled as C1 to C5) and four micropillar (labeled as P1 to P4) sample surfaces with periodic arrays of the microstructures, details in Experimental Section. **Figure** **2** illustrates the geometry design of the surfaces. Cone surfaces have square lattice and pillar surfaces have hexagonal lattice, and the varying parameter in both structure types is the spacing between the cones or pillars. The structure dimensions of the cone and pillar surfaces were determined with scanning electron microscopy (SEM), see **Table** **1** and Figures S1,S2 (Supporting Information). Tips of the cones have the aluminum mask remaining from the cone fabrication, see Figure 2d, and thus all the cone samples from C1 to C5 have rather consistent tip size and shape despite the very small length scales of the tips. The prepared cone surfaces were made superhydrophobic by coating them with an octyltrichlorosilane (OTS) self‐assembled monolayer coating and the pillar surfaces with a fluoropolymer coating, details in the Experimental Section. The prepared samples are superhydrophobic, and droplets roll off easily. More detailed wetting characterization will be presented later in this paper, as we will first focus on how stationary droplets sit on the surfaces.

**Table 1:** Dimensions of the cone and pillar surfaces were determined with SEM. Values are averages obtained from multiple cones/pillars at different locations on the surface (*n* = 8–12) and the error margins represent standard deviation.

Sample	Spacing ***s*** [µm]	Height ***h*** [µm]	Tip diameter *d*_tip_ [µm]	Tip half angle *β*_cone_ [°]
C1	0.96 ± 0.01	3.68 ± 0.02	0.096 ± 0.006	4.3 ± 0.4
C2	1.44 ± 0.01	3.72 ± 0.01	0.106 ± 0.007	4.7 ± 0.3
C3	1.92 ± 0.02	4.02 ± 0.01	0.111 ± 0.004	6.2 ± 0.3
C4	2.87 ± 0.02	4.16 ± 0.01	0.128 ± 0.017	3.0 ± 0.3
C5	3.84 ± 0.06	3.96 ± 0.06	0.108 ± 0.008	5.9 ± 0.6
P1	19.5 ± 0.1	35.9 ± 0.1	12.41 ± 0.03	–
P2	24.5 ± 0.1	37.7 ± 0.2	12.03 ± 0.03	–
P3	34.4 ± 0.1	38.9 ± 0.1	12.52 ± 0.05	–
P4	58.6 ± 0.1	40.4 ± 0.3	12.42 ± 0.08	–

### 2.1 How do Droplets Sit on Micropillar and ‐Cone Surfaces?

**Figure 3:**
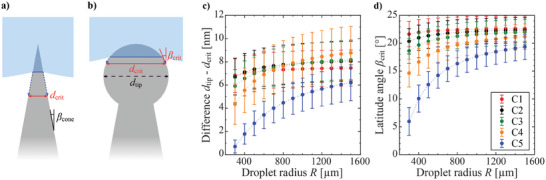
a) On ideally sharp cones, Equation 1 predicts droplet meniscus to sink to a level where the cone becomes wide enough (horizontal diameter *d*_crit_) to prevent the meniscus from further sinking. *β*_cone_ represents cone sidewall angle. b) On cones with blunt spherical tips, droplet meniscus can remain atop the tips with minimal vertical sinking. Equations (1), (2) predict the critical width *d*_crit_ and critical latitude angle *β*_crit_ needed to support droplet meniscus from further sinking. c) Difference of cone tip critical width *d*_crit_ calculated from Equations (1), (2) in relation to tip width *d*_tip_ obtained from SEM as function of droplet radius *R*. d) Critical latitude angle *β* calculated from Equation 1 and Equation 2 as function of droplet radius *R*. Data points in c) and d) represent average values, and error bars represent standard deviation of Equations (1), (2) solved via Monte Carlo simulations (*n*  =  3 × 10^6^), see Note S2 (Supporting Information). Dashed lines in c) and d) represent linear interpolation between data points and serve as guide for eye.

It is well known for the micropillar surfaces that water sits on top of the pillars (i.e., remains in the Cassie state), as long as droplets are placed gently on them.^[^ ^34^, ^35^ ^]^ On the contrary, droplets may sink into the cone surfaces and the amount depends on the dimensions of the cones as has been shown by Lecointre et al.^[^ ^5^ ^]^ Here, we can use a similar theoretical approach. For static droplets sitting on the periodic square array of micrometric‐sized cones, the contact line tension force opposes droplets sinking into the structure. The force scales with the length of the contact line, which grows the deeper the droplet sinks into the cone structure, as the cones become wider. The “critical width” of the cones (*d*_crit_, see **Figure** **3**a) enough to prevent droplets from further sinking in this case

(1) $$d_{\mathrm{crit}}=\;R\left|\cos\left(\theta_{\mathrm{adv}}-\beta \right)\right|\left(\sqrt{1+\frac{4s^{2}}{\pi R^{2}\cos^{2}\left(\theta_{\mathrm{adv}}-\beta \right)}}-1\right)$$

where *R* is the droplet radius, *s* is the cone spacing, *β* is the cone sidewall angle, and *θ*_adv_ is the advancing contact angle related to the surface chemistry of the cones, and Equation 1 is valid when *θ*_adv_ − *β* > 90° (see Note S1 Supporting Information for derivation of Equation 1). Equation 1 predicts that for all surfaces C1–C5 (*θ*_adv_ =  113°, corresponding OTS advancing contact angle, see Experimental Section), the value *d*_crit_ remains mostly below 100 nm for droplets with radius between 0.3 mm and 1.5 mm (Figure S3, Supporting Information).

The value of *d*_crit_ in this droplet size range is smaller than the tip diameter of the cones on surfaces C1–C5, meaning that the droplet meniscus would wet only the spherical cone tips. Equation 1 can also be applied to investigate how much the droplet meniscus would wet the spherical cone tips when taking into account that also *β* depends on the sinking depth. If assuming perfectly spherical tips (Figure 3b) *β* represents latitude angle of the spherical tip, and a simple relation between horizontal width *d* of the cone and its latitude angle *β* can be made,

(2) $$d\left(z\right)=d_{\mathrm{tip}}\;\cos\beta \left(z\right)$$

where *z* represents the sinking depth and *d*_tip_ the diameter of the spherical cone tip. Combining Equation 1 and Equation 2 yields that for all surfaces C1–C5, the critical diameter enough to prevent the sinking of the droplet meniscus is close to the tip diameter while the “critical slope” *β*_crit_ is ≈ 20°, see Figures 3c,d. Thus, the meniscus would wet the cones similarly as sketched in Figure 3b. In reality, the cone tips are not fully spherical but are somewhat flattened instead, which causes *d*_crit_ to be slightly wider than predicted by Equation 1 and Equation 2. Nearly the whole cone tip is wetted, so an approximation can be made that the droplet meniscus roughly wets a circular area with a diameter equivalent to the cone tip diameter.

**Figure 4:**
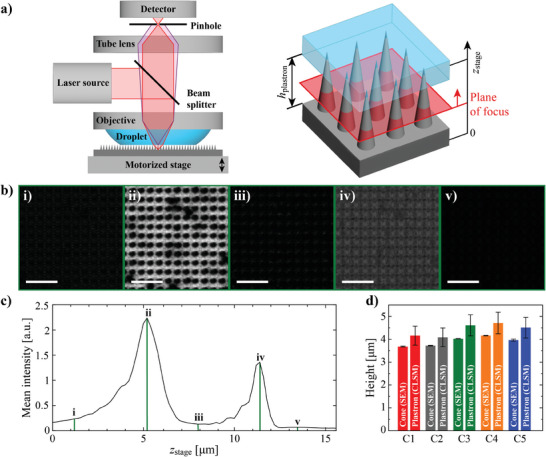
a) Schematics of the confocal microscopy setup. A droplet is attached to the lens and imaging occurs through the droplet that is in contact with the sample surface. Images are obtained with 0.2 µm spacing of focal planes starting from below the substrate surface (*z*_stage_ = 0) up to bulk water (*z*_stage_ = 15.6 µm). The pinhole limits the light collection only to the plane of focus (i.e., no light is collected from elsewhere). b) Example images i–v shown at different focal planes: ii is from the substrate surface bottom, iv is from the air–water interface and i, iii, and v are below the substrate surface, from the air layer, and from the bulk droplet, respectively, where no reflection of light occurs, thus forming dark images. The scale bar length is 5 µm. The images are from surface C2, other surfaces are presented in Figure S4 (Supporting Information). c) Mean brightness of the *z*‐stack frames at different focal planes. i–v mark the planes of images in b. Mean reflection intensities as a function of *z*_stage_ of other cone surfaces are shown in Figure S5 (Supporting Information). d) Comparison of cone height read from SEM images (left bars, data from Table 1) and measured plastron height (right bars). Values of plastron height represent average (*n* = 3) and error bars of the plastron height are ± 10%, see Notes S2,S3 (Supporting Information).

The theoretical consideration thus predicts that the droplets would not sink into the cone surfaces C1–C5. We decided to use confocal laser scanning microscopy (CLSM, details in Experimental Section) for an experimental check on whether droplets remain atop the cone tips or not. **Figure** **4**a shows the schematics of the experimental setup. By moving the microscope stage in the vertical direction, the CLSM can be focused accurately to different heights at the sample surface. There are two highly reflective planes in the system: the substrate bottom surface and the air‐water interface (called meniscus), see Figures 4b,c (images of all surfaces are shown in Figures S4,S5, Supporting Information). The distance between these two planes (i.e., the plastron height) can be read from the CLSM stage position. As light refracts at the air‐water interface, a correction

(3) $$h_{\mathrm{plastron}}\approx \Delta z_{\mathrm{stage}}\frac{n_{2}}{n_{1}}$$

based on Snell's law of refraction must be applied to convert the stage travel distance between the cone bottom surface and the meniscus levels (Δ*z*_stage_) to plastron height (*h*_plastron_). *n*_1_ and *n*_2_ are the refractive indices of water and air, respectively. Derivation and error estimation of Equation 3 are presented in Note S3 (Supporting Information).

For all surfaces C1–C5, the calculated plastron height is ≈10% higher than the cone height determined with SEM, see Figure 4d. Naturally, the plastron height cannot be larger than the cone height, but we ascribe this difference to the errors related to the CLSM measurements (see Notes S2,S3, Supporting Information). Nevertheless, these measurements show that water droplets either do not sink into the cone structures or sink only marginally (in the order of 100 nm) at maximum in any of the surfaces C1–C5. Therefore, the earlier theoretical estimation that the droplet meniscus remains at the tip vertices appears to be valid (additional discussion in Note S4, Supporting Information). The CLSM imaging also shows that the meniscus is rather flat (see Figure S5, Supporting Information) meaning that the meniscus does not sag significantly between the cones. This is in line with the theoretical prediction of the difference in critical latitude angle *β*_crit_ and advancing contact angle *θ*_adv_: their difference is nearly 90°, meaning that the meniscus is close to horizontal level when touching the cone tips.

### 2.2 Droplet Friction Characterization with Micropipette Force Sensor

**Figure 5:**
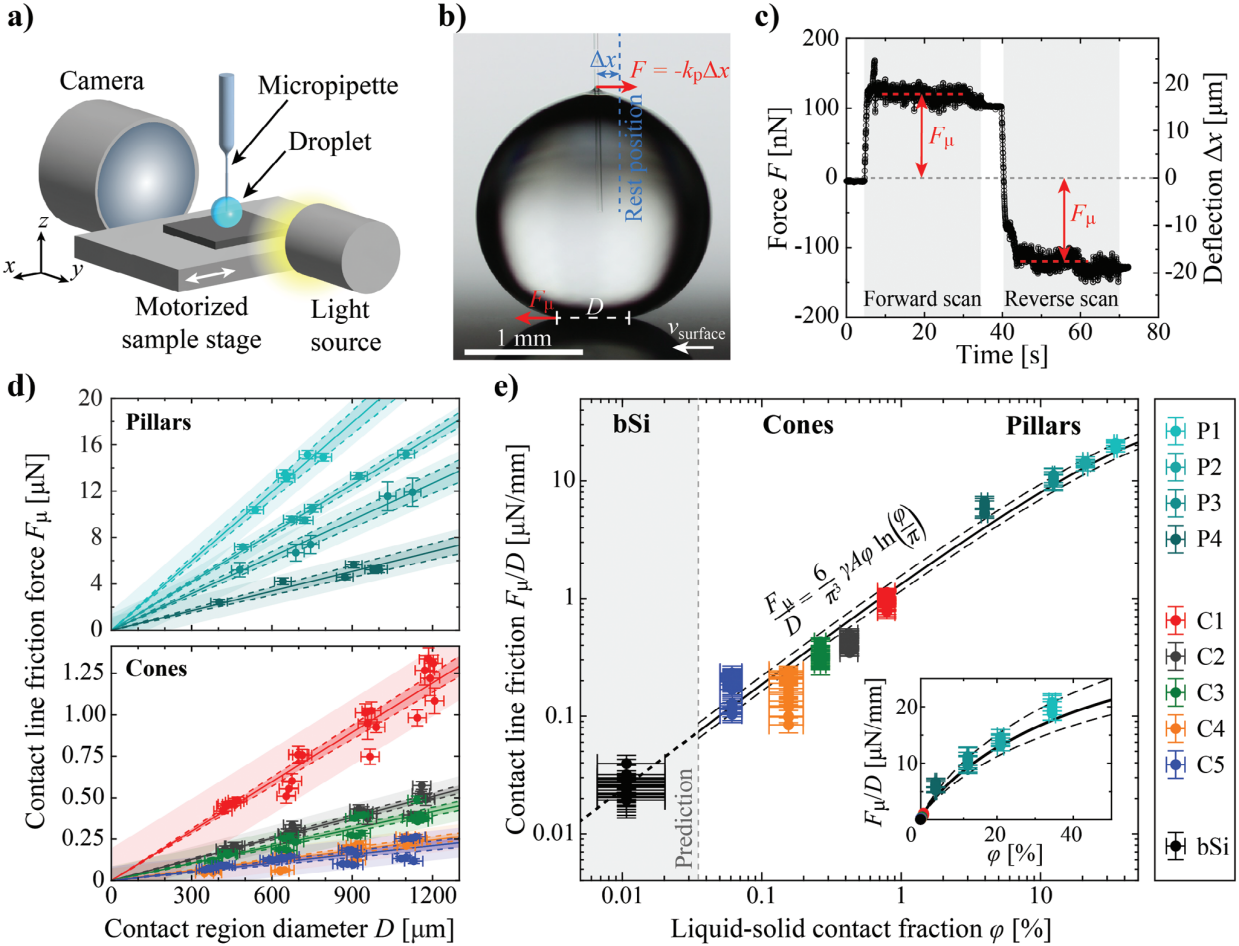
a) Schematics of the micropipette force sensor measurement setup. b) An example side view image of the droplet during a force scan. The dashed blue line represents the pipette rest position. The deflection is exaggerated in the image for visualization purposes, the real deflection is only tens of micrometers. c) A force versus time graph of a single scan. The force zero level is set to the average of forward and reverse scan forces determined in the regions marked by the red dashed lines. d) Measured CLF of pillar and cone surfaces as a function of droplet contact region diameter. The solid lines represent linear fits (through the origin) to the data. The dark shaded regions represent the 95% confidence intervals of the fit and the light shaded region the 95% prediction intervals of individual measurements. e) CLF force normalized by contact region diameter as a function of LS–CF. The solid black line represents a fit of Equation 6 to the cone and pillar data where ***ξ*** = 1.63. The dashed black lines represent fits to the cone data only (***ξ*** = 1.44, lower dashed line) and pillar data only (***ξ*** = 1.90, upper dashed line). The grey area represents an extrapolation of the fit (***ξ*** = 1.63) to the lower LS–CF regime. The LS–CF of the bSi surface is predicted with the fit (crossing of the fit and the reported average *F*_µ_/*D* value).^[^ ^41^ ^]^ In d and e, each data point represents a single MFS scan, and information on the error bars is in Note S2 (Supporting Information).

As mentioned earlier, the prepared cone surfaces C1–C5 and pillar surfaces P1–P4 are superhydrophobic, and the droplets on them are very mobile. Such surfaces are practically impossible to characterize accurately with the standard wetting characterization tool, contact angle goniometry, due to inaccuracy in determining the droplet baseline.^[^ ^24^, ^25^ ^]^ Backholm et al. developed a force‐based method that utilizes a micropipette force sensor (for measuring the droplet sliding friction on highly superhydrophobic surfaces,^[^ ^20^, ^28^ ^]^ and we use the same method in this work. The schematics of the measurement setup are illustrated in **Figure** **5**a, and a detailed description of the procedure can be found in Experimental Section.

In short, the droplet is dragged along the sample surface with the micropipette cantilever that has a known stiffness *k*_p_. The friction force causes the bending of the micropipette (see Figure 5b), and the amount of deflection Δ*x* relates to pulling force *F* via Hooke's law for spring force (*F*  =   − *k*_p_Δ*x*). Dragging the droplet on the sample surface from one point to another and back to the original location yields a force curve as shown in Figure 5c. As no other net forces are acting on the droplet in the horizontal direction, the pipette pulling force must relate directly to the sliding friction of the droplet during the constant motion phase. The sliding velocity is kept small to minimize internal viscous losses,^[^ ^36^, ^37^ ^]^ so the sliding friction practically equals to CLF force *F*_µ_, which relates to contact angle hysteresis via equation

(4) $$F_{\mu }=\frac{24}{\pi^{3}}\;\gamma D\left(\cos\theta_{\mathrm{rec}}-\cos\theta_{\mathrm{adv}}\right),$$

where γ is the liquid surface tension, *D* is the droplet contact region diameter and *θ*_adv_ and *θ*_rec_ are the advancing and receding contact angles, respectively.^[^ ^38^, ^39^, ^40^ ^]^ The factor 24π^−3^ holds when the droplet contact region remains circular.^[^ ^39^, ^40^ ^]^

The MFS experiments were performed for all surfaces C1–C5 and P1–P4, and Figure 5d shows the determined CLF force of each measurement. It is evident from the results that the friction force scales linearly with the droplet contact region diameter, as predicted by Equation 4. Droplet size independent contact line friction (*F*_µ_/*D*) of each sample surface is obtained via linear regression analysis. For the cones, the values range between 0.18–0.99 µN mm^−1^, corresponding to contact angle hysteresis of only ca. 5°–10° when assuming an advancing contact angle of 180°.^[^ ^34^ ^]^ The pillar surfaces have *F*_µ_/*D* values ranging between 5.8–19.9 µN mm^−1^, so significantly higher than those of the cone surfaces. On the other hand, in our earlier work, we found black silicon (bSi) surfaces (irregular conical microstructure, see Figure S6, Supporting Information) with the same OTS coating as used for C1–C5 to have the *F*_µ_/*D* value of 0.025 ± 0.005 µN mm^−1^,^[^ ^41^ ^]^ which is significantly lower than for those of the regular cone surfaces C1–C5.

### 2.3 Effect of Liquid–Solid Contact Fraction on Contact Line Friction

The main difference between these three types of surfaces is their topography which causes water to have different liquid–solid contact fractions on them. As discussed above, the water sits solely on the cone tips for all the surfaces C1–C5. Based on SEM images, the cone tip is a flattened sphere, and the LS–CF can be approximated as *φ* ≈ π*d*_tip_^2^/4*s* ^2^ (see Note S4 (Supporting Information) for a discussion on this approximation) and the values range between 0.06%–0.79%. The pillar surfaces have much higher LS–CF as the pillar tops are much wider, and the values range between 4.1%–36.8%. Reyssat & Quéré derived a relation between contact angle hysteresis and liquid–solid contact fraction when the liquid–solid contact points are circular and periodic:

(5) $$\cos\theta_{\mathrm{rec}}-\;\cos\theta_{\mathrm{adv}}=\frac{\xi }{4}\;\varphi \ln\left(\frac{\pi }{\varphi }\right)$$

where *ξ* is a parameter related to how strongly the liquid adheres to the solid (e.g., due to surface chemistry).^[^ ^11^ ^]^ Combining Equation 4 and Equation 5 gives a relation:

(6) $$\frac{F_{\mu }}{D}=\frac{6}{\pi^{3}}\;\gamma \xi \varphi \ln\left(\frac{\pi }{\varphi }\right)$$

and Figure 5e shows that cone and pillar surfaces indeed do follow the relation between *F*_µ_/*D* and φ when *ξ* = 1.63 despite the nearly three orders of magnitude difference between the LS–CF of the surfaces. In addition, parameter *ξ* seems not to be significantly sensitive for the microstructure vertex size as pillar diameter is ca. 500‐fold compared to cone tip diameter, nor it is significantly sensitive for the surface chemistry, as *θ*_adv_/*θ*_rec_ on smooth surfaces of OTS and fluoropolymer are 113°/106°^[^ ^41^ ^]^ and 111°/85°,^[^ ^42^ ^]^ respectively. Fitting Equation 6 separately for cones yields *ξ* = 1.44 and correspondingly for pillars *ξ* = 1.90, leading only to a minor difference of the fit as visualized with the dashed lines in Figure 5e.

The bSi surface has a random surface geometry in the sub‐micrometer length scale that prevents direct calculation or measurement of the LS–CF. Despite Equation 6 being derived for periodic contact points, it should also apply for randomly spaced contact points as long as the spatial variations in the LS–CF remain in the micrometer length scales. Therefore, Equation 6 also enables predicting the LS–CF of the bSi surface, and with *ξ* = 1.63, a droplet on the bSi surface would have an average LS–CF of only 0.007%–0.020%. This is almost an order of magnitude less than for the sparsest cone surface C5 which has the lowest LS–CF of the cone surfaces, and such an LS–CF fraction has not been reported for any surfaces earlier.

## 3 Conclusion

To conclude, we have explored how droplet sliding friction relates to liquid–solid contact fraction on regular micropillar and ‐cone surfaces. A theoretical consideration was presented to show that the cone surfaces used in this work have dense enough arrays of microcones so that they can fully support droplets and only the cone tip vertices become wetted. Confocal laser scanning microscopy imaging also supports this finding by showing that there is no observable difference in the plastron height in relation to the cone height. Therefore, the liquid–solid contact fraction of the microcone surfaces used in this work can be calculated based on the tip geometry, similar to the micropillar surfaces. Droplet contact line friction measurements with the micropipette force sensor technique revealed extremely small friction values down to 0.2 µN mm^−1^ for the sparsest microcone surface C5 and showed that the contact line friction and liquid–solid contact fraction relates to each other via a model developed earlier by Reyssat and Quéré.^[^ ^11^ ^]^ The model was also found to simultaneously fit the contact line friction of microcone as well as micropillar surfaces that have liquid–solid contact fractions extending almost over three orders of magnitude from 0.06% to 36.8%. The good fit of the model enables us to use it for estimating even smaller liquid–solid contact fraction such as for bSi surfaces with random conical microstructures. However, we note that irregular, anisotropic, or re‐entrant surface geometries may alter scaling between droplet friction and liquid–solid contact fraction, calling for additional research on this topic. Nevertheless, our work deepens the knowledge of how droplets behave on conical microstructures, which may help in designing surfaces for applications such as anti‐icing where minimal contact between water and the surface is beneficial.

## 4 Experimental Section

### Microcone Sample Preparation

(100) silicon substrates were coated with AR‐P 6200 e‐beam resist after which electron beam lithography (EBL, Vistec EPBG5000pES) was used to pattern periodic arrays of circles with a 100 nm nominal diameter. After the resist development, a nominal 20 nm thick aluminum film was deposited on the substrates by e‐beam evaporation (Instrumentti Mattila). A liftoff step was then carried out in AR 600–71 remover to define aluminum disks on the Si surface. The substrates were then glued onto aluminum oxide coated carrier wafers using photoresist and etched using a cryogenic inductively coupled plasma reactive ion etching process (ICP‐RIE, Oxford Instruments Plasmalab 100) at −110 °C with a mixture of SF_6_ and O_2_ as the process gas.^[^ ^41^ ^]^ After the etch, the samples were removed from the carrier wafers by dissolving the photoresist in acetone, after which the samples were immersed in isopropyl alcohol to remove any remaining acetone residue.

The fabricated microcone surfaces were then cleaned and activated with 30 min UV‐O_3_ treatment (BioForce Nanosciences). Directly after the treatment, they were transferred into an atomic layer deposition (ALD) reactor (Savannah S200, Veeco) pre‐heated to 60 °C. The reactor was pumped down to vacuum (ca. 10 Pa base pressure), after which the reactor chamber was purged with 20 sccm nitrogen flow. Next, ca. 1 nm film of aluminum oxide (Al_2_O_3_) was grown on the microcone surface with trimethyl aluminum (TMA) and water ALD process for 10 cycles. The pulse and purge times for both TMA (Volatec Oyj) and water (Milli‐Q) were 15 ms and 30 s, respectively, and nitrogen flow was kept at 20 sccm throughout the process. The idea of the thin aluminum oxide layer was to provide a fresh and conformal, OH group rich surface for the SAM growth.

After the aluminum oxide film growth was finished, the samples were further kept in the ALD reactor for the octyltrichlorosilane (OTS) growth step, which is described in more detail in earlier work.^[^ ^41^ ^]^ In short, the nitrogen flow was first turned off and the reactor chamber was isolated by closing the “stop valve” between the vacuum pump and the chamber. Then ca. 50 Pa dose of OTS (97%, Sigma–Aldrich) was applied into the reactor chamber. The reactor was periodically evacuated from HCl that had been generated as a reaction by‐product and from the small amount of ambient air leaked into the reactor due to the reactor's natural leakage rate. In total, the OTS exposure lasted 48 h. After the exposure, the reactor was vented, and samples were removed from the reactor and stored in ambient air in a closed plastic box before sample characterization. The OTS on a flat Al_2_O_3_ surface is shown to yield advancing and receding contact angles of 113° and 106°, respectively.^[^ ^41^ ^]^ All five microcone surfaces with different cone spacing were treated simultaneously to ensure equal SAM growth on all of them.

### Micropillar Sample Preparation

(100) silicon substrates with 510 nm thermal oxide were patterned with photolithography (AZ 5214E photoresist and MA6 mask aligner, Karl Suss). The oxide was etched by reactive ion etching, 25 sccm CHF_3_, 25 sccm Ar, 200 mTorr pressure, 30 W power, and 14 min etching time (Plasmalab 80+, Oxford Instruments). The photoresist was then stripped by ultrasonicating in acetone and using oxygen plasma. The micropillars were etched by cryogenic deep reactive silicon etching, 40 sccm SF_6_, 6 sccm O_2_, 8 mTorr pressure, 1050 W ICP power, 3W platen power, −110 °C temperature, 18 min etching time (Plasmalab system 100, Oxford Instruments). After etching, the oxide mask was stripped in BFH.

The prepared micropillars were coated with a PECVD fluoropolymer, 100 sccm CHF_3_, 250 mTorr pressure, 50 W power, and 5 min deposition time (Plasmalab 8+. Oxford Instruments). On a flat Si substrate, fluoropolymer coating was earlier shown to yield advancing and receding contact angles of 111° and 85°, respectively.^[^ ^43^ ^]^

### Scanning Electron Microscopy (SEM)

Scanning electron microscopy was performed with Sigma VP (Zeiss). For the cone surfaces, a 4 nm iridium coating was sputtered (Leica EM ACE600) to minimize charging of the surface. No conductive coating was applied on the pillar surfaces. Imaging of all surfaces was performed using the In‐lens detector with an acceleration voltage of 2.00 kV.

### Confocal Laser Scanning Microscopy (CLSM)

Confocal microscopy was performed with an upright Leica SP8 equipped with a 25x/0.95 water immersion objective (HC Fluotar L, Leica). The microcone substrates were placed on the XYZ motorized microscope stage. A droplet of Milli‐Q water was dispensed to the lens of the microscope, after which the stage was lifted to bring the sample surface in contact with the droplet hanging from the lens. Next, the sample was imaged at 79 different stage heights with a step height of 0.2 µm (i.e., a z‐stack was recorded). The imaged area was 46.6 × 46.6 µm with a stack total height of 15.6 µm and laser light with a wavelength of 488 nm was used for the imaging. The imaging was repeated three times at different locations on the sample surface.

### Micropipette Force Sensor (MFS)

A detailed description of the MFS setup was described earlier.^[^ ^20^, ^28^ ^]^ In short, a micropipette with a ca. 30 mm long cantilever (outer diameter ca. 36 µm) was prepared from glass capillaries (inner/outer diameter = 0.75/1 mm, TW100‐6, World Precision Instruments) using a micro‐pipette puller (PN‐31, Narishige) and a micropipette forge (MF‐900, Narishige). The cantilever stiffness (*k*_p_ = 6.82 ± 0.1 µN mm^−1^) was calibrated by placing the micropipette horizontally, dispensing a MilliQ‐water droplet (density 1.00 g cm^−3^) to the end of the pipette and monitoring the droplet volume and pipette deflection due to the weight of the droplet with a side view camera (Canon EOS 90D, Canon MP‐E macro lens at ca. 4X magnification, resulting in 3.03 µm/pixel and field of view of 5.8 × 3.3 mm). The stiffness calibration was repeated 5 times, and the reported pipette spring constant was the average of the results and standard deviation was used as an error estimation of the stiffness.

The droplet sliding friction measurements with MFS were performed by positioning the pipette vertically over a motorized XYZ stage and the sample surface on the stage below the pipette tip. A droplet was dispensed to the end of the pipette tip such that the upper part of the droplet is in contact with the pipette while its lower part rests on the sample surface. The setup was then let to stabilize for 30 s to dampen possible vibrations caused during the droplet dispensing, after which the droplet was dragged with the micropipette along the surface by moving the stage first in positive *x*‐direction (0.1 mms^−1^ sample stage velocity, 3.0 mm distance) and then in the negative *x*‐direction with the same velocity and distance. The dragging event and pipette deflection were recorded with the side view camera (same settings as with pipette stiffness calibration). The amount of pipette deflection was analyzed from the recordings using MATLAB. The pipette zero position (i.e., no deflection) was set to the middle point between average deflections during forward and backward scans. The CLF force was then calculated based on pipette stiffness and deflection from the zero position: *F*_µ_ = −*k*_p_ Δ*x*, and its average value was calculated over the time periods when the droplet was moving (see Figure S7a, Supporting Information). The droplet contact region diameter was read from the videos by looking for the narrowest point between the droplet and its reflection, see Figures S7b–d (Supporting Information). The average contact region diameter was read over the same time periods as was used to calculate the average CLF. The MFS experiments were repeated with several different droplet sizes ca. 1–10 µl at different locations on the sample surfaces.

The MFS experiments of micropillar surfaces were performed otherwise similarly as for the cone surfaces, but the scans were performed with a stiffer micropipette (*k*_p_ = 138.2 ± 1.1 µN mm^−1^) and to forward direction only. The pipette zero position was obtained by dragging the droplet in the *y*‐direction (i.e., toward and out of the camera) before the scan, which effectively removes any pipette deflection in the *x*‐direction. After that, the droplet was dragged to the positive *x*‐direction (0.1 mms^−1^ sample stage velocity, 5.0 mm distance).

### Statistical Analysis

Dimensions of the cones and pillars were analyzed from SEM images. Several (*n* = 8–12) cones/pillars were selected from random locations of the sample, and their sizes were determined from the images. Presented dimensions in the article are average ± standard deviation.

Droplet friction and contact region diameter data were obtained from MFS scans. The scans contain signal noise and possible systematic errors, on which the error analysis was based, see Note S2 (Supporting Information). Linear fits of CLF force versus contact region diameter data and related confidence and prediction intervals were performed with MATLAB using functions “lsqcurvefit” and “nlpredci”. The fit of Equation 6 to CLF versus LS–CF data was performed in MATLAB using the function “lsqcurvefit”.

Plastron height was determined with CLSM from three different locations and the presented value represents the average (*n* = 3). The error margins were ± 10% and they were based on simulations, see details in Notes S2,S3 (Supporting Information).

Cone tip critical width *d*_crit_ and slope *β*_crit_ were calculated based on Equations (1), (2). Due to the complexity of the pair of equations, the error analysis was performed via Monte Carlo simulation (*n*  =  3  × 10^6^) method by modelling cone spacing *s* and cone tip diameter *d*_tip_ as random distributions with their average and standard deviation values determined from the SEM images of the cone surfaces, see details in Note S2 (Supporting Information).

## Conflict of Interest

The authors declare no conflict of interest.

## Supporting information



Supporting Information

## Data Availability

The data that support the findings of this study are available from the corresponding author upon reasonable request.
