# Supplementary material for: Droplet Friction on Superhydrophobic Surfaces Scales With Liquid‐Solid Contact Fraction
Source: Small. 2024 Sep 17;21(7):2405335. doi: 10.1002/smll.202405335 (PMC11840469; doi:10.1002/smll.202405335)
Supplement: Supplementary file 1 — Supporting Information [file SMLL-21-2405335-s001.docx]

Supporting Information

Droplet friction on superhydrophobic surfaces scales with liquid-solid contact fraction

Sakari Lepikko, Valtteri Turkki, Tomi Koskinen, Ramesh Raju, Ville Jokinen, Mariia S. Kiseleva, Samuel Rantataro, Jaakko V.I. Timonen, Matilda Backholm, Ilkka Tittonen, and Robin H. A. Ras*

This file includes

- Supplementary Figures S1 – S8
- Supplementary Notes 1-4

Supplementary Figures


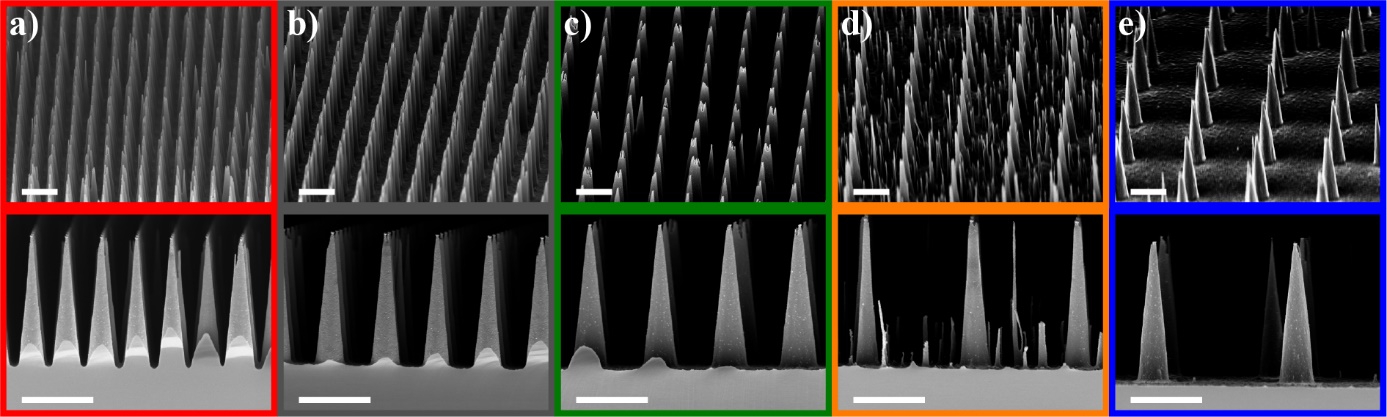


**Figure S1.** SEM images of the microcone surfaces, **a)** C1, **b)** C2, **c)** C3, **d)** C4, and **e)** C5. Top row shows an image at 60° tilt from the surface normal and bottom row shows a sideview image. Scale bar is 2 µm in all images. Surface C4 shows more additional random spike formation during the ICP-RIE process than the other surfaces but the height of those spikes is mostly well below the cone height and thus does not affect the overall wetting performance of the surface.


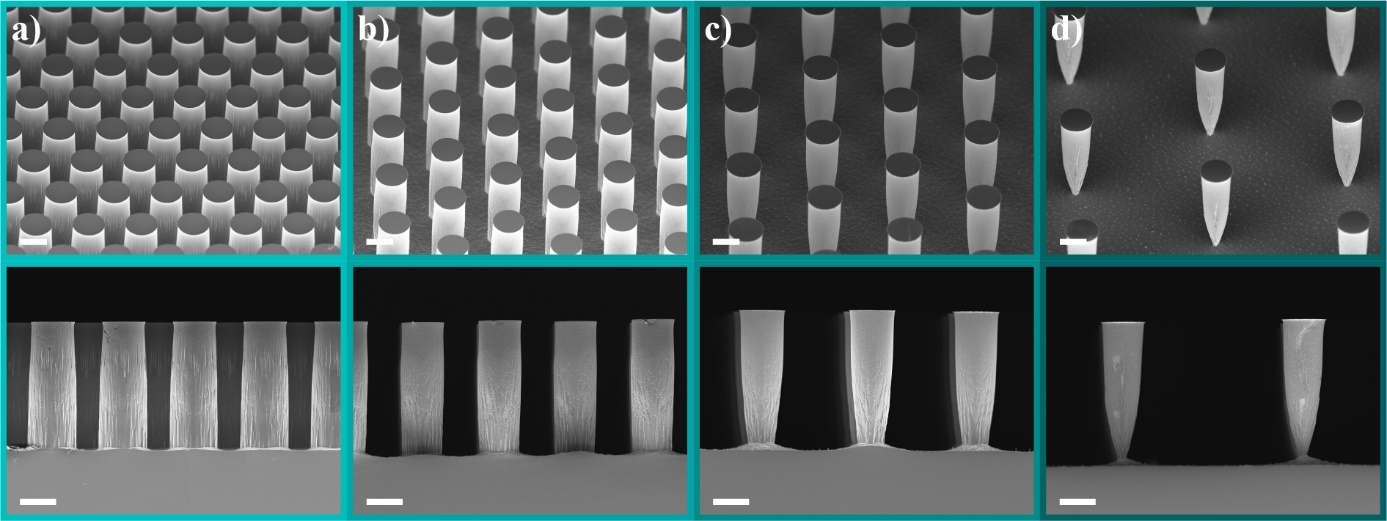


**Figure S2.** SEM images of the micropillar surfaces, **a)** P1, **b)** P2, **c)** P3, and **d)** P4. Top row shows an image at 45° tilt from the surface normal and bottom row shows a sideview image. The scale bar is 10 µm in all images.


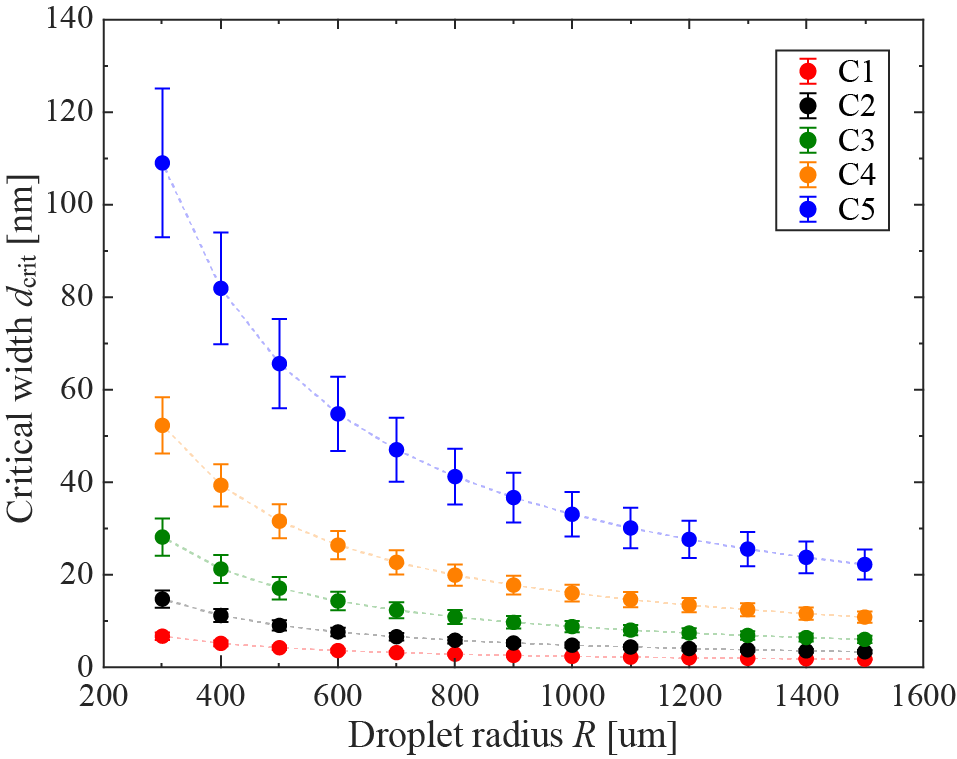


**Figure S3**. Calculation of the critical cone tip diameter $d_{\mathrm{crit}}$ at sinking depth $z$ as function of droplet radius $R$ using Equation 1 (Equation S12) when assuming that cones have sharp tips. Error bars are obtained via Monte Carlo simulations, see Supplementary Note 2. Dashed lines represent linear interpolation between data points and serve as guide for eye.


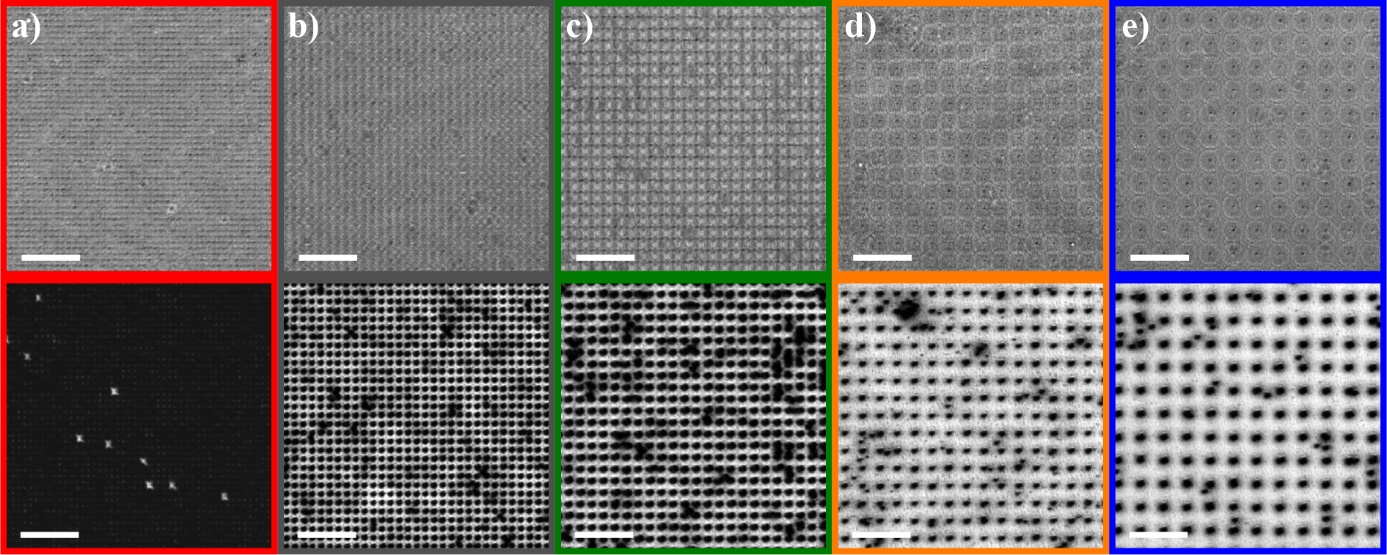


**Figure S4.** XY planes of the CLSM images of surfaces **a)** C1, **b)** C2, **c)** C3, **d)** C4, and **e)** C5. The top row represents XY plane images acquired from the air-water interface reflection and the bottom row represents XY plane images acquired from the substrate surface reflection. The substrate surface plane of the sample C1 is determined based on the brightness of the reflection from the spots that are missing cones. The image bit depth is reduced from 16 to 8, and image brightness and contrast are adjusted separately for each image for best image clarity. The scale bar length is 10 µm in all images.


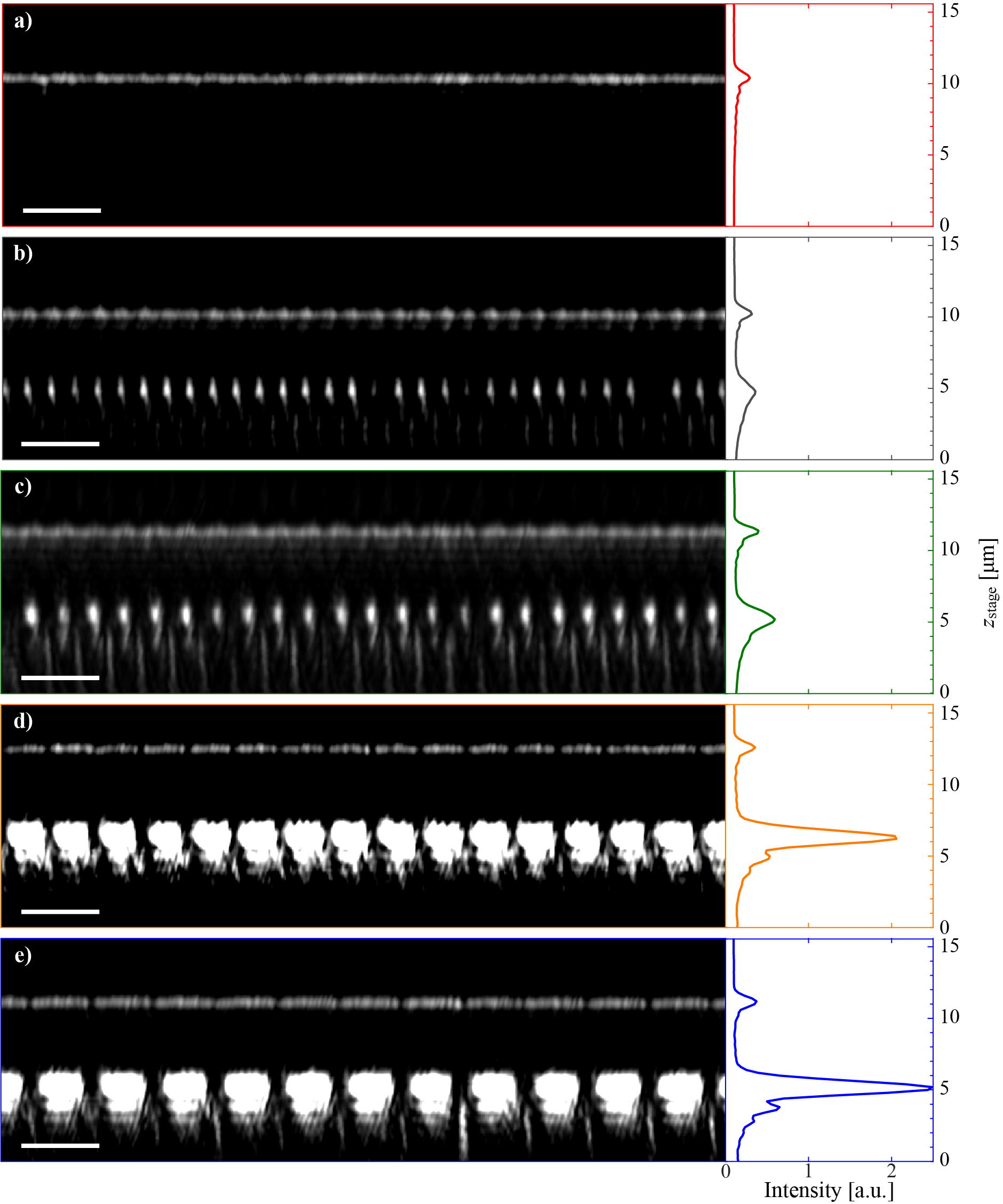


**Figure S5.** XZ planes of CLSM z-stacks of surfaces **a)** C1, **b)** C2, **c)** C3, **d)** C4, and **e)** C5. The graphs on the right show the mean intensity of the XY plane as function of the $z$-coordinate. The lower peak and the bright features in the images around $z_{\mathrm{stage}}$ = 5 µm are due to the reflection from the substrate surface. The peak and the narrow line around $z_{\mathrm{stage}}$ = 12 µm in the images are due to the reflection from the air water interface. The image bit depth is reduced from 16 to 8, and image brightness and contrast are adjusted separately for each image for best image clarity. The scale bar length is 5 µm in all images.


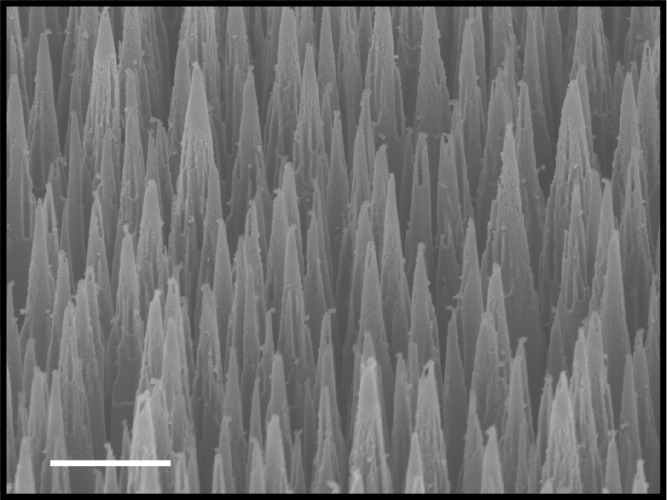


**Figure S6.** SEM image of the black silicon surface. Scale bar is 2 µm. Image obtained from ref^[1]^ with permission.


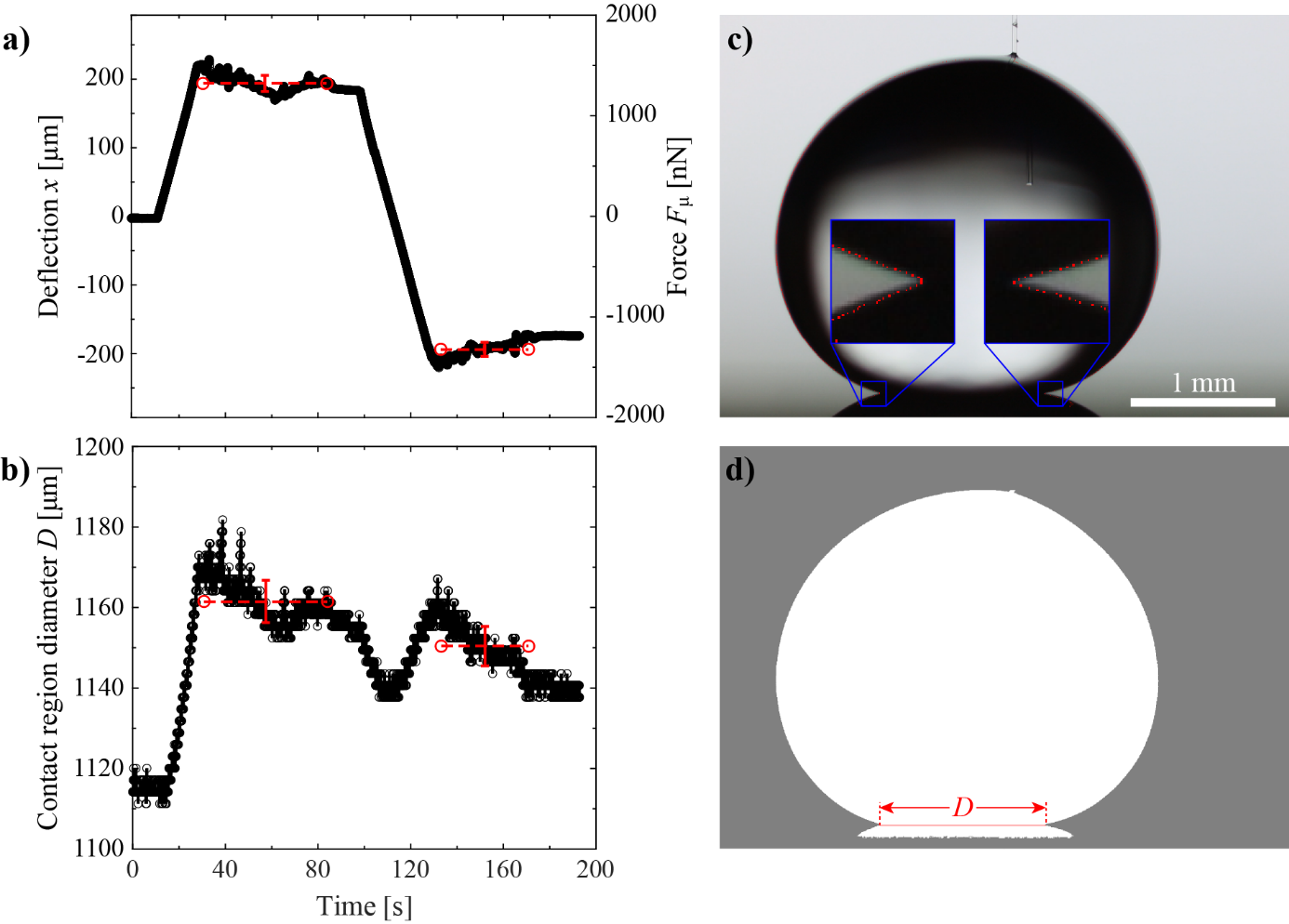


**Figure S7.** **a)** Pipette deflection and **b)** droplet contact region diameter during a MFS scan. The red dotted lines mark regions where the droplet moves during the forward and reverse scans. The height of the lines represents the average deflection/contact region diameter in these regions and the error bars standard deviation. **c)** A snapshot of a moving droplet during a MFS scan and **d)** binary image of it obtained via thresholding. The edge of the thresholded region is marked with red line into panel c. The inset images in c shows 5x magnifications to the advancing (right inset) and receding (left image) triple phase contact points.


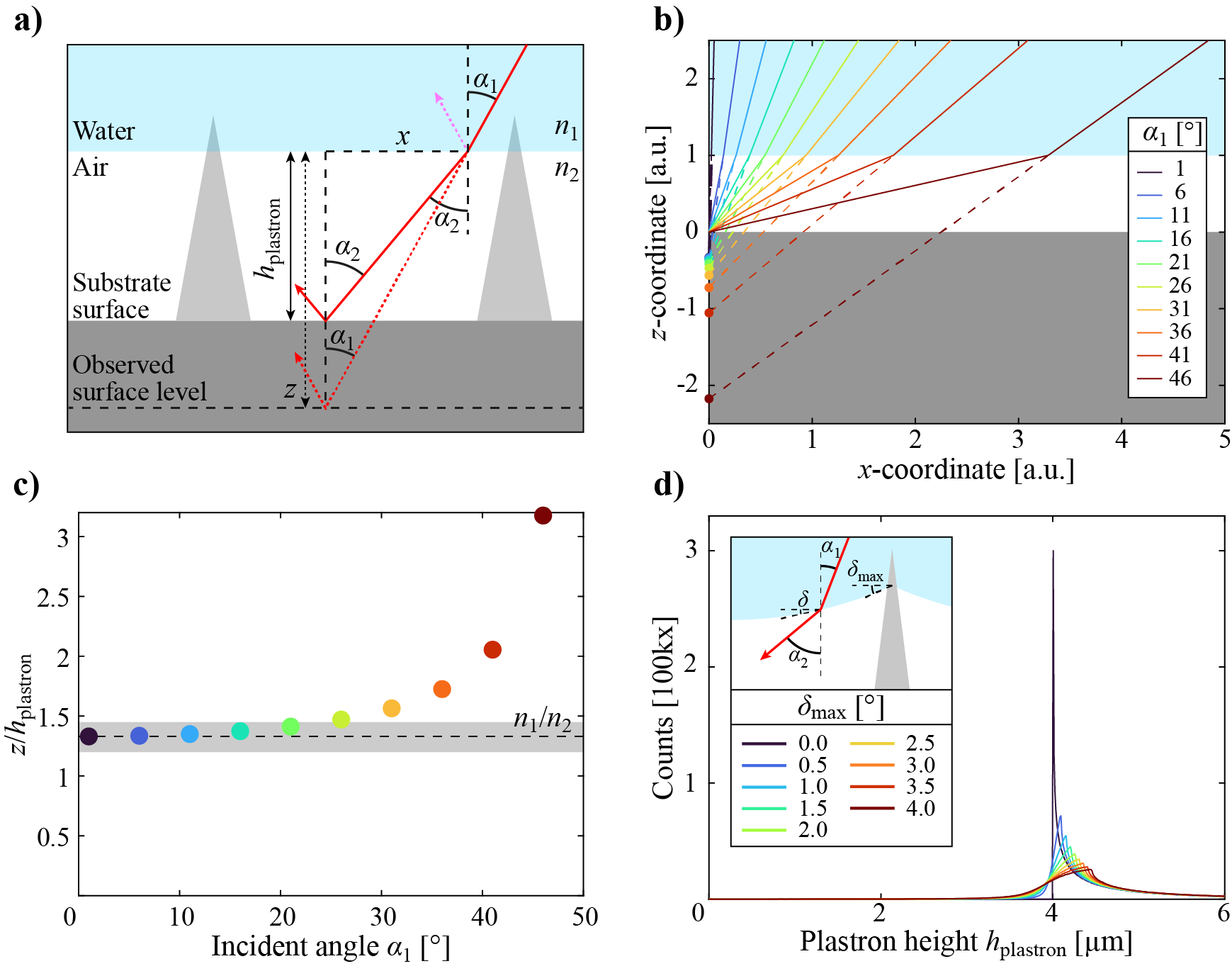


**Figure S8.** **a)** Propagation of light at the sample surface. Light refracts at the air-water interface and reflects from the substrate surface. $\alpha_{1}$ represents incident angle, $\alpha_{2}$ refraction angle, $n_{1}$ refractive index of water, $n_{2}$ refractive index of air, $x$ the horizontal distance that light travels inside the plastron before encountering the substrate bottom surface, $h_{\mathrm{plastron}}$ the plastron height, and $z$ the apparent plastron height when ignoring refraction of the incident beam. **b)** Refraction of light at air-water interface with different incident angles $\alpha_{1}$. Dotted lines represent light rays when refraction is ignored. **c)** Relative difference of $z$ and $h_{\mathrm{plastron}}$ for different incident angles $\alpha_{1}$. The dotted line represents ratio $n_{1}/n_{2}$ and the shaded region represents 10% difference margin. **d)** Monte Carlo simulations ($3 \times{10}^{6}$ per simulated line) of plastron height calculation with varying incident angle $\alpha_{1}$ and tilt of the air-water interface $\delta$. The counts represent observations of plastron height at 10 nm increments. The inset shows how refraction of light depends on incident angle $\alpha_{1}$ and the local tilt of the air water interface. The value in the legend shows the maximum tilt $\delta_{\max}$ in each of the simulations. See details of the simulations in Supplementary Note 3.

**Supplementary Notes**

Supplementary Note 1. Details of cone tip critical diameter calculation

*Model for ideal sharp cones*

Lecointre *et al*. presented in their publication a formula for droplet sinking depth into hexagonal lattice of sharp circular cones^[2]^. Following the same reasoning, we present here a similar formula that is valid for square lattice of cones and for droplets (radius $R$) that are significantly larger than the cone spacing $s$ ($R\gg s$), but smaller than the capillary length $\lambda_{c}$ ($R<\lambda_{c})$. Considering a situation where four cones set the corners of the unit cell of the lattice, the contact line perimeter $D$ of the droplet is simply

|  | $D=4\times\frac{\pi}{2}b\left( z \right)=2\pi b\left( z \right)$ | (S1) |
| --- | --- | --- |

where $b\left( z \right)$ is the cone radius a distance $z$ from the top of the cone (simply related as $b\left( z \right)=z\tan\beta$). The force $F_{\mathrm{ST}}$ caused by the surface tension of this contact line perimeter is

|  | $F_{\mathrm{ST}}=\gamma D\cos\left( \theta-\beta\right)$ | (S2) |
| --- | --- | --- |

where $\gamma$ is surface tension of the liquid, $\theta$ is the contact angle of the chemical coating on a smooth surface, and $\beta$ is the cone half angle. By dividing the force by the area of the liquid air interface between the cones in the unit cell ($A= s^{2}-\pi b^{2}$) we get a pressure $P_{\mathrm{ST}}\left( z \right)$, which in equilibrium balances the Laplace pressure $P_{L}=2\gamma/R$ of the droplet, i.e., ${P_{L}=P}_{\mathrm{ST}}\left( z \right)$. Combining everything so far together and solving out $d_{\mathrm{crit}}=2b\left( z \right)$ yields

|  | $d_{\mathrm{crit}}=R\left\vert\cos\left( \theta-\beta\right) \right\vert\left( \sqrt{1+\frac{4s^{2}}{\pi R^{2}\cos^{2} \left( \theta-\beta\right)}}-1 \right)$ | (S3) |
| --- | --- | --- |

There are some aspects that need to be considered when using the obtained Equation S3. Firstly, Equation S3 is valid when $\theta-\beta>$ 90°. For $\theta-\beta<$ 90° the droplet would spontaneously sink into Wenzel state regardless of the cone tip size. Secondly, the value of $\theta$ corresponds best to the advancing contact angle. If a droplet would start sinking, its meniscus would advance down the surfaces of the cones, which is best described by the advancing contact angle. Therefore, it can be set that $\theta=\theta_{\mathrm{adv}}$ in Equation S3. Finally, the $d_{\mathrm{crit}}$ depends on the droplet radius $R$, which is illustrated in Figure S3. In the regime $s\ll R<\lambda_{c}$, $d_{\mathrm{crit}}$ is always smaller than the cone tip diameter of surfaces C1-C5, and thus the LS-CF is independent on droplet radius (when $s\ll R<\lambda_{c}$) on those surfaces.

*Model for cones with spherical tips*

It is possible to use similar reasoning as described above for ideal sharp cones for cones with spherical tips (as presented in main text Figure 2b). The main difference is that the value of angle $\beta$ depends on the sinking depth $z$. For spheres, the angle $\beta(z)$ can be related to the sphere horizontal radius $b\left( z \right)$ via a simple relation $2b\left( z \right)=d_{\mathrm{tip}}\cos\beta(z)$. Therefore, a pair of equations

| $d_{\mathrm{crit}}=d_{\mathrm{tip}}\cos\beta_{\mathrm{crit}}=R\left\vert\cos\left( \theta_{\mathrm{adv}}-\beta_{\mathrm{crit}} \right) \right\vert\left( \sqrt{1+\frac{4s^{2}}{\pi R^{2}\cos^{2} \left( \theta_{\mathrm{adv}}-\beta_{\mathrm{crit}} \right)}}-1 \right)$ | (S4) |
| --- | --- |

can be used to solve the combination of minimum horizontal width $d_{\mathrm{crit}}$ and minimum slope $\beta_{\mathrm{crit}}$ at which the droplet meniscus is able remain without further sinking. Equation S4 has similar validity limitations as Equation S3. Firstly $\theta_{\mathrm{adv}}-\beta_{\mathrm{crit}}>$ 90°, other solutions lead to sinking below the spherical tip. Secondly, Equation S4 also assumes $s\ll R<\lambda_{c}$.

**Supplementary Note 2. Error estimation**

*Cone tip diameter needed to support a droplet*

The pair of critical cone tip width and latitude angle needed to support the droplet from sinking is based on theoretical considerations presented in main text Equation 1 and Equation 2. The considerations involve use of multiple experimental values, thus the errors were estimated based on a Monte Carlo simulation technique. Firstly, cone spacing, cone tip diameter and advancing contact angle were assumed to follow a normal distribution with mean and standard deviation obtained from SEM images (cone spacing and tip diameter) and contact angle measurements (advancing contact angle). Randomly generated values of $s$, $d_{\mathrm{tip}}$ and $\theta_{\mathrm{adv}}$ were then used as initial values for solving Equation 1 and Equation 2 in total for $n=3\times{10}^{6}$ times, yielding average values for cone (tip) critical diameter $d_{\mathrm{crit}}$ and tip critical latitude angle $\beta_{\mathrm{crit}}$ and related standard deviation values are used as error estimators for $d_{\mathrm{crit}}$ and $\beta_{\mathrm{crit}}$.

*Plastron height in CLSM*

Plastron height was determined from three different locations for surfaces C1-C5 with CLSM. The standard deviation of the determined plastron height is 0.03 µm, which is much less than the systematic errors related to the plastron height determination with Equation 3 (see Supplementary Note 3). Therefore, the error of the plastron height inside the cone structures can be estimated based on the difference of simulations with perfectly flat air-water interface and interface with maximum tilt $\delta_{\max}$ = 2°, giving $\Delta h_{\mathrm{plastron}}$ ≈ 10%. The microscope stage and the refractive index of water are assumed accurate.

*Liquid-solid contact fraction*

Determination of the LS-CF is based on the cone and pillar tip diameter determined from SEM images. The error related to LS-CF stems from variations between individual cones and pillars and possible systematic errors related to the measurements and to the assumption of the wetted area of the tip (especially for cones, see Supplementary Note 4). Since the systematic errors are difficult to quantify, and the effect would be of the same magnitude and direction for all the samples, they are excluded from the error analysis. Thus, the error of the LS-CF is based on the standard deviation of the cone dimensions reported in Table 1 in the main text, and the error of the LS-CF is obtained by error propagation. For cones, the error is

|  | $\Delta\varphi_{\mathrm{cone}}=\frac{\pi d_{\mathrm{tip}}}{2s^{2}}\Delta d_{\mathrm{tip}}+\frac{\pi{d_{\mathrm{tip}}}^{2}}{{2s}^{3}}\Delta s$ | (S5a) |
| --- | --- | --- |

and for pillars the error is

|  | $\Delta\varphi_{\mathrm{pillar}}=\frac{\pi d_{\mathrm{tip}}}{\sqrt{3}s^{2}}\Delta d_{\mathrm{tip}}+\frac{\pi{d_{\mathrm{tip}}}^{2}}{\sqrt{3}s^{3}}\Delta s$ | (S5b) |
| --- | --- | --- |

*Droplet sliding friction with micropipette force sensor*

The friction force of a droplet moving on a surface is determined from the average pulling force of the pipette over certain scan distance during which the droplet motion is steady. During the pulling, the pipette vibrates causing noise to the friction data, see Figure S7a. This vibration is the largest error source in the measurements, and we measure it by taking the standard deviation of the pipette deflection over the scan distance. The micropipette stiffness has also a small inaccuracy, and we assume that the standard deviation of the repeated stiffness calibration (see Methods) represents this inaccuracy. We assume that the pipette deflection determination from the image has high accuracy. Thus, the total error of the friction force by error propagation is

|  | $\Delta F_{\mu}=x\Delta k_{p}+k_{p}\Delta(\Delta x)$ | (S6) |
| --- | --- | --- |

The droplet contact region diameter during an MFS has two error sources. Firstly, the contact region diameter varies during a scan, see Figure S7b. This variation we model via standard deviation over the scan region. Secondly, the contact region diameter is read from the recorded video (see Figure S7c-d), and we assume accuracy of ±5 pixels, corresponding to ±15 µm, in locating the contact region extremes. The total error of the contact region diameter is calculated to be the sum of these two error components.

The error related to the $F_{\mu}/D$value we obtain by error propagation:

|  | $\Delta\left( F_{\mu}/D \right)=\frac{\Delta F_{\mu}}{D}+\frac{F_{\mu}\Delta D}{D^{2}}$ | (S7) |
| --- | --- | --- |

Supplementary Note 3. Details of CLSM and its accuracy analysis

Due to the difference of refractive indices of water and air, the distance between the substrate surface level and the droplet bottom appears incorrectly, as is shown in Figure S8a. With basic geometry,

|  | $\left\{ \begin{aligned} \frac{x}{z}=\tan\alpha_{1} \\ \frac{x}{h_{\mathrm{plastron}}}=\tan\alpha_{2} \end{aligned}\Rightarrow h_{\mathrm{plastron}}= z\frac{\tan\alpha_{1}}{\tan\alpha_{2}} \right.$ | (S8) |
| --- | --- | --- |

where all symbols are as defined in Figure S8a. There is no solution independent from at least one of the refraction angles $\alpha$, meaning that light coming at different incident angles have always different $z$. However, for small angles it can be approximated that $\tan\alpha\approx\sin\alpha$, which together with Snell’s law for refraction yields

|  | $h_{\mathrm{plastron}}\approx z\frac{n_{2}}{n_{1}}$ | (S9) |
| --- | --- | --- |

where $n_{1}$ and $n_{2}$ are the refractive indices of water and air, respectively.

The objective lens used in this work has a numerical aperture (NA) of 0.95, meaning that the maximum incident angle $\alpha_{1}$ able to pass through the lens is

|  | $NA=n_{1}\sin\alpha_{1}\Rightarrow\alpha_{1}\approx46^{\circ}$ | (S10) |
| --- | --- | --- |

This means that the objective lens collects light with incident angles from 0° to 46°, and the approximation $\tan\alpha\approx\sin\alpha$ is not accurate over the whole range of incident angles. Calculating $z$ as a function of the incident angle $\alpha_{1}$ reveals that the approximation in Equation S9 is valid (error < 10%) until ca. $\alpha_{1}$ = 25° while beyond it the relative error increases rapidly, see Figure S8b‑c. As a result, the observed reflection intensity peak from the substrate surface shifts towards smaller $z$, meaning that the plastron height appears larger than predicted by Equation S9.

It has been assumed above that the air-water interface is perfectly flat, as Figure S5 does not show any observable sagging of the air-water interface between the cones. However, even a small tilt of the air-water interface ($\delta$) affects the refraction of the light significantly. Figure S8d shows a set of MATLAB® Monte Carlo simulations ($n$ = 3 x 10^6^ in each simulation) of Equation S8 for the plastron height $h_{\mathrm{plastron}}$. In the simulations, the incident angle $\alpha_{1}$ variers between 0° and 46° (uniform distribution) and the refraction angle $\alpha_{2}$ is obtained via equation

|  | $\alpha_{2}=\sin^{-1} \left( \frac{n_{1}}{n_{2}}\sin\left( \alpha_{1}+\delta\right) \right)-\delta$ | (S11) |
| --- | --- | --- |

where $\delta$ describes the tilt of the air-water interface at the location where the incident light ray hits the interface, see the inset of Figure S8d. $\delta$ varies between ${-\delta}_{\max}$ and $\delta_{\max}$ (uniform distribution, $\delta_{\max}$ is in range of 0° to 4° for different simulations). Plastron height is set to 4 µm in the simulations. Without any tilt of the air water interface, the calculated plastron height is close to the set plastron height, but even at minor sagging of the air-water interface (causing non-zero tilt of air-water interface) the peak shifts towards larger z-values. The peak also broadens significantly with increasing $\delta_{\max}$. Such large broadening of the reflection intensity peak is not observed with real CLSM measurements performed in this work, indicating that the air-water interface of real droplets on the conical surfaces is relatively flat, well below maximum tilt of 2°. This basically caps the plastron thickness determination error to ca. 10%, meaning that sinking of the droplet into the cone structure is marginal, in the order of 100 nm at maximum.

**Supplementary Note 4. Additional discussion on liquid-solid contact fraction of microcone surfaces**

The calculation of the LS-CF of water on the cone surfaces C1-C5 has been based on both theoretical and experimental background. As presented in main text, the force from the contact line tension of water at the cone tips opposing the sinking is such high that water should remain atop the cone oblate-shaped tips. Due to the oblate shape of the tips, the wetting occurs approximately over the whole tip area (contact line sets close to the equator of the cone tip), meaning that LS-CF can be approximated as $\varphi\approx\pi{d_{\mathrm{tip}}}^{2}/4s^{2}$. This approximation may cause some inaccuracy in the analysis of the relation between LS-CF and CLF, but the nature of the error should be systematic and magnitude relatively small in comparison to differences of LS-CF of the different surfaces.

CLSM measurements provide experimental support for the theoretical calculations, although the CLSM accuracy is not enough to exclude some small amount of sinking (maximum in the order of some hundreds of nanometers) that could affect the LS-CF water has on those surfaces. However, additional support for the theoretical calculations can be also read from the CLF results. Had there been sinking below the cone tips, the amount of sinking should have been different for the different cone surfaces C1-C5 due to the different cone spacing. Namely, most sinking should have occurred for the sparsest cone surface C5 and least for the densest C1, thus the LS-CF of the sparse cone surfaces should differ most from the approximated value. However, for none of the surfaces C1-C5, CLS does not differ significantly from the prediction obtained from Equation 6, indicating that there should not be any significant sinking of the meniscus below the cone tips. Only the sparsest surface C5 has on average slightly higher CLF than predicted by the model, so some minor, practically insignificant amount of sinking could have occurred on it. Therefore, the used approximation $\varphi\approx\pi{d_{\mathrm{tip}}}^{2}/4s^{2}$ can be considered valid for the cone surfaces C1-C5.

**Supplementary References**

[1] S. Lepikko, Y.M. Jaques, M. Junaid, M. Backholm, J. Lahtinen, J. Julin, V. Jokinen, T. Sajavaara, M. Sammalkorpi, A.S. Foster, and R.H.A. Ras, *Nat. Chem.* **2024**, 16, 506.

[2] P. Lecointre, S. Laney, M. Michalska, T. Li, A. Tanguy, I. Papakonstantinou, D. Quéré, *Nat. Commun.* **2021**, *12*, 3458.
